# Uric Acid and Uric Acid Index in Predicting Coronary Artery Disease, Cerebrovascular Events, and Mortality: A Sex‐Stratified Cohort Study

**DOI:** 10.1002/clc.70376

**Published:** 2026-06-15

**Authors:** Ali Rezaee, Mobina Imannezhad, Farzam Kamrani, Mohsen Moohebati, Bahram Shahri, Hedieh Alimi, Habibollah Esmaily, Hanie Mahaki, Mohsen Mehrabzadeh, Alireza Heidari‐Bakavoli, Majid Ghayour‐Mobarhan, Susan Darroudi

**Affiliations:** ^1^ Student Research Committee, Faculty of Medicine Mazandaran University of Medical Sciences Sari Iran; ^2^ Department of Nutrition, Faculty of Medicine Mashhad University of Medical Sciences Mashhad Iran; ^3^ Department of Cardiovascular, School of Medicine Mashhad University of Medical Sciences Mashhad Iran; ^4^ Department of Biostatistics, School of Health Mashhad University of Medical Sciences Mashhad Iran; ^5^ Social Determinants of Health Research Center Mashhad University of Medical Sciences Mashhad Iran; ^6^ Vascular and Endovascular Surgery Research Center Mashhad University of Medical Sciences Mashhad Iran; ^7^ Metabolic Syndrome Research Center, School of Medicine Mashhad University of Medical Sciences Mashhad Iran

**Keywords:** cerebrovascular events, coronary artery disease, uric acid, uric acid index

## Abstract

**Aim:**

Recent studies have highlighted the predictive value of serum uric acid (SUA), fasting blood glucose (FBG), and triglycerides (TG) for cardiovascular diseases (CVDs). A newly developed uric acid (UA) index exhibits useful, but further validation is required to establish its superior predictive power over SUA. This study aimed to compare the predictive capabilities of the UA index and SUA in CVD, coronary artery disease (CAD), and stroke.

**Methods:**

This study was conducted based on the Mashhad Stroke and Heart Atherosclerotic Disorders (MASHAD) study. SUA and the UA index (Ln [TG (mg/dL) × SUA (mg/dL) × FBG (mg/dL)/2]) were calculated at baseline. After 10 years, the incidence of the outcomes including CAD, cerebrovascular events, and death by CAD or cerebrovascular events were evaluated.

**Results:**

SUA showed a modest but significant association with CAD in the total population (HR = 1.064, 95% CI: 1.010–1.121, *p* = 0.020), but was not associated with stroke or mortality, and showed no significant associations in gender‐stratified analyses. In contrast, UAI demonstrated a strong and consistent association with CAD across all groups. Each unit increase in UAI was associated with a 54.1% higher risk of CAD (HR = 1.541, 95% CI: 1.407–1.689, *p* < 0.001), with stronger effects in women. UAI was also significantly associated with increased mortality risk, but not with stroke. ROC analysis showed that UAI had modest but consistently better discriminative ability than SUA for predicting CAD and mortality (AUC: 0.632 vs. 0.561).

**Conclusion:**

In conclusion, UAI outperformed SUA in predicting CAD and mortality, particularly in women, and may serve as a more robust biomarker for cardiovascular risk assessment.

## Graphical abstract

Uric acid index, integrating SUA, TG, and FBG, outperforms serum uric acid alone in predicting coronary artery disease and mortality over 10 years, with stronger effects in women, highlighting its value as a more robust cardiovascular risk biomarker.

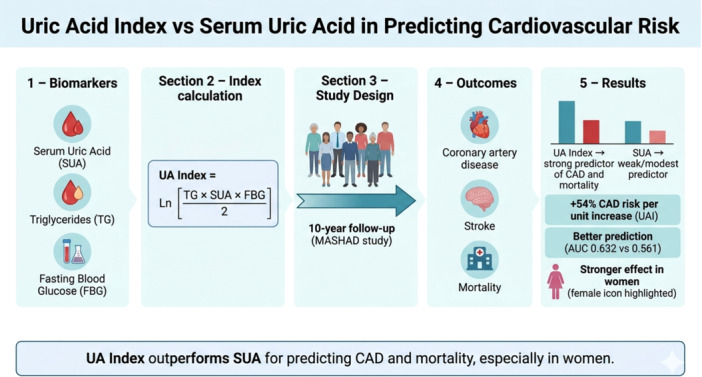

## 1 Introduction

Uric acid (UA) is the final product of purine metabolism in higher animals, including humans and great apes. Under normal conditions, the body balances UA production and excretion. When this balance is disrupted, it results in hyperuricemia [1, 2]. While some studies emphasize the antioxidant properties of serum uric acid (SUA), others underscore its pro‐oxidant effects [3, 4]. However, elevated SUA levels can lead to inflammation and oxidative stress in the body [5]. Furthermore, a randomized, parallel‐controlled study involving 176 patients with type 2 diabetes and asymptomatic hyperuricemia found that allopurinol effectively reduced SUA levels, improved insulin resistance (IR), decreased serum high‐sensitivity C‐reactive protein (hs‐CRP) levels, lowered carotid intima‐media thickness, and mitigated the progression of atherosclerosis [5]. Therefore, SUA may serve as an additional contributor to cardiovascular disease (CVD) risks.

In addition to UA, high fasting blood glucose (FBG) and elevated serum triglyceride (TG) levels can lead to atherosclerosis and contribute to CVD through oxidative stress and inflammation [6, 7, 8]. As a result, Rojas‐Humpire et al. developed a new index related to UA that considers not only SUA levels but also FBG and TG [9]. Despite being a recent development, this index has already revealed a significant positive correlation between the risk of CVD and cognitive impairment, leading to new possibilities for research and potential treatment approaches [10].

The burden of CVD is projected to increase sharply in Iran from 2005 to 2025 [11, 12]. In 2010, CVD was the leading cause of death in Iran [13]. Therefore, preventing CVD is a critical strategic priority for the country's health system [14, 15]. The cross‐sectional nature of the Rojas‐Humpire study made it impossible to establish a cause‐and‐effect relationship. It is important to note that the study's small sample size (291 participants) is a limitation. Our study aims to address this gap in current research by comparing the correlation between UA and UA index with coronary artery disease (CAD), cerebrovascular events, and death by CAD or cerebrovascular events while also considering the striking role of gender in this context. Through this approach, we aim to gain valuable insights that will contribute to developing gender‐specific healthcare strategies.

## 2 Methods

### 2.1 Study Population

This study was based on the Mashhad Stroke and Heart Atherosclerotic Disorders (MASHAD) Study, which consisted of 9704 individuals [16]. Healthy individuals aged between 35 and 65 years were included in the study. Clustered sampling was randomly conducted from three regions of Mashhad city, in the northeast of Iran. Participants with a history of CVD, CAD, and having cancer or autoimmune diseases were excluded. The MASHAD study is a prospective cohort that started in 2010 and was followed up until 2020, with follow‐up every 3 years by phone. After a 10‐year period, 1060 CVD events, including CAD, cerebrovascular events, and deaths caused by CAD or cerebrovascular events, were documented. All 9704 participants with complete baseline and follow‐up data were included in the final analysis.

### 2.2 Ethics

The study was approved by the ethics committee of Mashhad University of Medical Sciences (MUMS) (IR.MUMS.IRH.REC.1403.133), and informed consent was obtained from all participants. Additionally, the study was conducted in accordance with the Declaration of Helsinki.

### 2.3 Anthropometric and Biochemical Measurements

Blood samples were taken from individuals after 12 h of fasting to measure FBG, lipid profile (total cholesterol, TG, low‐density lipoprotein [LDL], and high‐density lipoprotein [HDL]), and SUA levels using the auto analyzer. Weight and height were measured according to the standard protocol. Body mass index (BMI) was calculated through the formula: weight (kg)/(height)^2^ (m^2^). Also, waist circumference (WC) was measured with tape in the narrowest area between the iliac crest and the lowest rib. Additionally, systolic blood pressure (SBP) and diastolic blood pressure (DBP) were measured twice by a standard sphygmomanometer from the left arm while participants were in a sitting position after rest. Calculation of the UA index is based on a specific formula defined as [9]:

UAI=Ln[TG(mg/dl)×SUA(mg/dl)×FBG(mg/dl)/2]

### 2.4 Outcome Assessments

A 12‐lead ECG was conducted by a cardiologist, and additional tests such as stress echocardiography, radioisotope scan, angiography (≥ 50% of stenosis in at least one major coronary artery), CT angiography, and exercise tests were performed as necessary to confirm or rule out the presence of CAD [17]. Cerebrovascular events were defined as ischemic stroke, intracerebral hemorrhage (ICH), and transient ischemic attack (TIA), according to established definitions from the American Heart Association/American Stroke Association [18].

Mortality due to CAD or Cerebrovascular events was confirmed using International Classification of Diseases (ICD‐10) codes. The events were categorized into four groups: no event, CAD, cerebrovascular events, and death caused by CAD or cerebrovascular events.

### 2.5 Statistical Analysis

Data normality was assessed using the Kolmogorov−Smirnov test. Continuous data were expressed as mean ± standard deviation (SD), while categorical data were presented as frequency (%). Qualitative data were compared using the chi‐square test, and quantitative data were compared using a sample *T*‐test. The association between SUA and UA index and study outcomes was determined using a Cox regression model, reported as hazard ratios (HRs) ± 95% confidence intervals (CIs). The analysis was adjusted for age, marital status, education level, job status, smoking status, BMI, SBP, and DBP. Receiver operating characteristic (ROC) analysis has been conducted to compare the UA and uric acid index (UAI) to predict events. Data analysis was performed using SPSS (version 26; SPSS Inc., Chicago, IL). A *p* value of less than 0.05 was considered statistically significant.

## 3 Results

A total of 9704 individuals participated in the study, the mean age of participants was 48.07 ± 8.25 years, with males being slightly older than females (48.84 ± 8.42 vs. 47.56 ± 8.07 years, *p* < 0.001). Regarding socio‐demographic characteristics, the majority of participants were married, with a significantly higher proportion among males compared to females. Educational level differed significantly between genders, with males more frequently having higher education levels, while females were predominantly in the low education category (*p* < 0.001). Employment status also showed marked differences, with most males being employed and most females being unemployed (*p* < 0.001). Smoking was more prevalent among males, whereas females were more likely to be nonsmokers (*p* < 0.001) (Table 1).

**Table 1:** Demographic data of the study population.

		Total	Male (*N* = 3023)	Female (*N* = 4538)	*p* value
Age, years		48.07 ± 8.25	48.84 ± 8.42	47.56 ± 8.07	< 0.001
Marriage status	Single	59 (0.6)	19 (0.5)	40 (0.7)	< 0.001
	Married	9039 (93.1)	3834 (98.7)	5204 (89.4)	
	Divorced	134 (1.4)	17 (0.4)	117 (2)	
	Widow	472 (4.9)	14 (0.4)	458 (7.9)	
Educational level	Low	5288 (54.5)	1609 (41.4)	3679 (63.3)	< 0.001
	Moderate	3348 (34.5)	1600 (41.2)	1748 (30.1)	
	High	1061 (10.9)	676 (17.4)	385 (6.6)	
Job status	Employee	3605 (37.2)	2860 (73.6)	745 (12.8)	< 0.001
	Unemployed	5145 (53)	330 (8.5)	4815 (82.8)	
	Retired	950 (9.8)	694 (17.9)	256 (4.4)	
Smoking status	Nonsmoker	6654 (68.6)	2236 (57.6)	4418 (75.9)	< 0.001
	Ex‐smoker	958 (9.9)	589 (15.2)	369 (6.3)	
	Current smoker	2092 (21.6)	1060 (27.3)	1032 (17.7)	
BMI, kg/m^2^		27.89 ± 4.74	26.35 ± 4.14	28.92 ± 4.84	< 0.001
WC, cm		95.22 ± 12.04	93.69 ± 11.04	96.24 ± 12.56	< 0.001
SBP, mmHg		121.86 ± 19.12	122.38 ± 17.18	121.52 ± 20.31	0.023
DBP, mmHg		79.16 ± 11.78	80.04 ± 10.63	78.57 ± 12.45	0.001
FBG, mg/dL		90.7 ± 39.32	91.86 ± 37.62	93.26 ± 40.4	0.08
Cholesterol, mg/dL		191.35 ± 39.14	186.78 ± 37.8	194.39 ± 39.7	< 0.001
TG, mg/dL		142.55 ± 92.44	149.4 ± 99.76	137.99 ± 86.86	< 0.001
LDL, mg/dL		116.52 ± 35.3	113.46 ± 34.5	118.56 ± 35.68	< 0.001
HDL, mg/dL		42.86 ± 9.94	39.84 ± 9.25	44.87 ± 9.88	< 0.001
SUA, mg/dL		4.66 ± 1.4	5.27 ± 1.44	4.25 ± 1.21	< 0.001
UA index		10.08 ± 0.79	10.24 ± 0.77	9.97 ± 0.78	< 0.001
Event	No event	8644 (89.1)	3371 (86.8)	5273 (90.6)	< 0.001
	CAD	784 (8.1)	362 (9.3)	422 (7.3)	
	Cerebrovascular events	91 (0.9)	36 (0.9)	55 (0.9)	
	Death by CAD or Cerebrovascular events	185 (1.9)	116 (3)	69 (1.2)	

Abbreviations: BMI, body mass index; CAD, coronary artery disease; DBP, diastolic blood pressure; HDL, high‐density lipoprotein; LDL, low‐density lipoprotein; SBP, systolic blood pressure; SUA, uric acid; TG, triglyceride; UI, uric acid index; WC, waist circumference.

In terms of clinical characteristics, females had significantly higher BMI and WC compared to males (*p* < 0.001). Males had slightly higher systolic and diastolic blood pressure. Analysis of lipid profile showed that females had higher total cholesterol, LDL, and HDL levels, while males had higher TG levels (*p* < 0.001). SUA levels were significantly higher in males (5.27 ± 1.44 mg/dL) compared to females (4.25 ± 1.21 mg/dL), and the UAI was also higher in males (*p* < 0.001) (Table 1).

During the follow‐up period, cardiovascular outcomes were recorded. The prevalence of CAD was higher in males compared to females (9.3% vs. 7.3%), while cerebrovascular events were relatively similar between genders. Mortality due to CAD or cerebrovascular events was higher in males, at 3% (Table 1).

**Figure 1:**
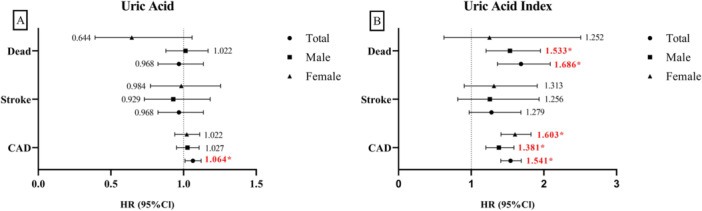
Association of serum uric acid (A) and uric acid index (B) with cardiovascular outcomes (CAD, cerebrovascular events, and death by CAD or cerebrovascular events) stratified by sex. Cox regression analysis has been done; data adjusted for age, marital status, educational level, job status, smoking status, BMI, SBP, and DBP. The red bold values with a star are statistically significant, *p* < 0.05.

Based on the Cox regression analysis, SUA showed a modest but statistically significant association with CAD in the total population (HR = 1.064, 95% CI: 1.010–1.121, *p* = 0.020). However, SUA was not significantly associated with stroke or mortality outcomes (*p* > 0.05). In gender‐stratified analysis, SUA did not show significant associations with any outcomes in either males or females (Figure 1A). In contrast, the UAI demonstrated a strong and significant association with CAD across all groups. In the total population, each unit increase in UAI was associated with a 54.1% higher risk of CAD (HR = 1.541, 95% CI: 1.407–1.689, *p* < 0.001). This association remained significant in both males (HR = 1.381, 95% CI: 1.201–1.588, *p* < 0.001) and females (HR = 1.603, 95% CI: 1.410–1.823, *p* < 0.001), with a stronger effect observed in women. Regarding mortality, UAI was significantly associated with increased risk in the total population (HR = 1.686, 95% CI: 1.361–2.088, *p* < 0.001) and in males (HR = 1.533, 95% CI: 1.204–1.952, *p* = 0.001), while no significant association was observed in females. Although UAI showed a positive trend with stroke risk, this association did not reach statistical significance in any group (Figure 1B).

In addition to the Cox regression findings, we calculated the discriminative ability of SUA and the UAI for predicting cardiovascular outcomes using ROC curve analysis.

**Figure 2:**
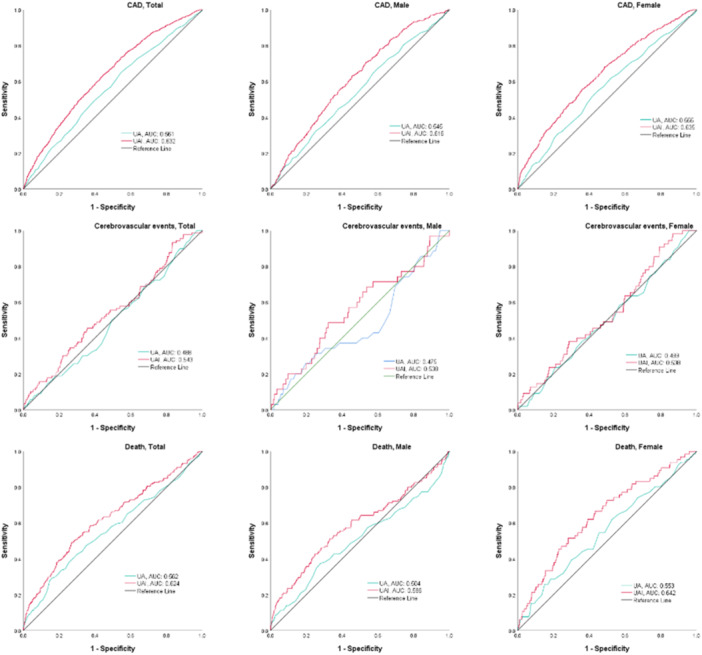
Receiver operating characteristic (ROC) curves comparing the predictive performance of serum uric acid and uric acid index for cardiovascular outcomes (CAD, cerebrovascular events, and death by CAD or cerebrovascular events).

For CAD, the UAI demonstrated modest but consistently better predictive performance than SUA in all groups. In the total population, the area under the curve (AUC) for UAI was 0.632, compared to 0.561 for SUA. Similarly, in males, UAI showed higher discrimination (AUC = 0.616) than SUA (AUC = 0.545), and in females, UAI (AUC = 0.635) outperformed SUA (AUC = 0.555) (Figure 2). Regarding cerebrovascular events, both SUA and UAI showed relatively weak predictive ability. However, UAI still performed slightly better than SUA. In the total population, AUC values were 0.543 for UAI and 0.488 for SUA. In males, UAI had an AUC of 0.538 compared to 0.475 for SUA, while in females, UAI demonstrated an AUC of 0.538 versus 0.499 for SUA (Figure 2). For mortality due to CAD or cerebrovascular events, UAI again showed superior predictive ability. In the total population, AUC for UAI was 0.624, compared to 0.562 for SUA. Among males, UAI had an AUC of 0.586, while SUA showed an AUC of 0.504. In females, UAI demonstrated the highest predictive performance (AUC = 0.642) compared to SUA (AUC = 0.553) (Figure 2). Overall, the ROC analysis confirmed that the UAI consistently has better discriminative power than SUA alone for predicting CAD and mortality outcomes, with the strongest predictive performance observed in women. However, both markers showed limited predictive ability for cerebrovascular events.

## 4 Discussion

In this cohort study, SUA and the UAI were measured at baseline. The incidence of CAD, cerebrovascular events, and death due to CAD or cerebrovascular events was evaluated over a 10‐year follow‐up, with stratification by gender. Our findings demonstrated that while SUA showed only a modest association with CAD in the total population, UAI exhibited a significantly stronger and more consistent association with both CAD and mortality outcomes.

SUA was significantly associated with CAD only in the overall population, with no significant associations observed in gender‐stratified analyses or for other outcomes such as stroke and mortality. These findings suggest that SUA alone may have limited utility as an independent predictor of cardiovascular outcomes, particularly when sex‐specific differences are considered. This is consistent with previous studies reporting inconsistent or weak associations between SUA and certain cardiovascular endpoints, especially stroke. Evidence regarding the relationship between SUA and stroke remains conflicting [19]. A study including 12,739 stroke cases reported a positive association between SUA levels and improved ischemic stroke prognosis [20], suggesting a potential neuroprotective role of SUA in acute settings [21, 22]. However, a more recent meta‐analysis found no significant association between SUA levels and stroke prognosis [23].

In contrast, UAI demonstrated a robust and consistent association with CAD across all groups, including both males and females, with a stronger effect observed in women. This finding highlights the added value of incorporating metabolic components such as TGs and FBG into UA‐related risk assessment [20]. The stronger association observed in women may be attributed to sex‐specific hormonal and metabolic differences. This may be due to lower baseline SUA levels and greater sensitivity to metabolic disturbances in women, making relative increases more clinically meaningful [12, 24, 25]. Additionally, previous evidence indicates that elevated FBG and the triglyceride‐glucose (TyG) index are associated with an increased risk of ischemic stroke [25, 26]. Although our findings showed no significant association between SUA and stroke, the inclusion of glucose and TGs in UAI may provide a more comprehensive reflection of metabolic risk.

CVD encompasses a wide range of disorders affecting the heart and vasculature, including CAD, heart failure, arrhythmias, and stroke [27]. Atherosclerosis plays a central role in these conditions, with plaque formation being a key determinant of future cardiovascular events [27, 28]. Cerebral ischemia results from insufficient blood supply to the brain, leading to tissue damage and infarction [29], with atherosclerosis being the primary underlying cause [29, 30].

From a mechanistic perspective, several biological pathways may explain the observed associations. Elevated SUA contributes to oxidative stress, inflammation, and endothelial dysfunction [31]. During purine metabolism, xanthine oxidase generates reactive oxygen species, which impair nitric oxide bioavailability and vascular function [32, 33]. Elevated SUA has been widely recognized as a contributor to cardiovascular complications [31], and pharmacological reduction of SUA using xanthine oxidase inhibitors such as allopurinol has demonstrated anti‐ischemic effects in clinical trials [34, 35]. Furthermore, large‐scale meta‐analyses have reported a dose‐response relationship between SUA levels and cardiovascular mortality [36], and hyperuricemia has been linked to other cardiovascular risk factors including hypertension, obesity, and metabolic syndrome [37].

However, recent evidence suggests that combining SUA with metabolic parameters enhances predictive accuracy. The interaction between TGs and glucose contributes to IR and metabolic dysregulation, which in turn influences UA metabolism [10, 36]. Studies have demonstrated synergistic effects between SUA and metabolic indices such as the TyG index in predicting cardiovascular outcomes [38]. This interaction may be mediated through shared pathways involving lipid metabolism and blood pressure regulation [39, 40]. Additionally, elevated fasting glucose in combination with SUA has been associated with vascular changes such as increased carotid intima‐media thickness [30, 41]. In line with these findings, Rojas‐Humpire et al. reported that the UAI is a stronger predictor of CVD compared to SUA alone [9]. Our results, based on a larger cohort and longitudinal design, further support this observation.

Importantly, ROC curve analysis in our study confirmed the superior discriminative ability of UAI compared to SUA in predicting CAD and mortality outcomes. The predictive performance of UAI was consistently higher across all groups, with the strongest performance observed in females. These findings support the concept that composite indices incorporating multiple metabolic factors provide more accurate cardiovascular risk stratification than single biomarkers [42, 43].

Sex‐specific differences may further explain these findings. Hormonal factors, particularly estrogen, play a significant role in UA metabolism. Previous studies have shown that SUA levels are influenced by reproductive hormones, with inverse relationships observed with estradiol and progesterone levels [44, 45]. Lower baseline SUA levels in women may render them more sensitive to metabolic disturbances, thereby amplifying the predictive value of UAI in this group [46].

Regarding cerebrovascular events, neither SUA nor UAI demonstrated strong predictive performance, and no significant associations were observed in regression analyses. This may indicate that UA‐related markers are less relevant for cerebrovascular outcomes compared to coronary outcomes. Previous literature in this area remains inconsistent, likely due to variations in study design and population characteristics [37, 47].

The superior performance of UAI may be explained by its ability to capture multiple interrelated pathophysiological pathways [48]. While SUA contributes to oxidative stress and endothelial dysfunction, TGs and glucose reflect underlying IR and metabolic imbalance [49]. These factors act synergistically to promote inflammation, lipid accumulation, and vascular injury, ultimately accelerating atherosclerosis and increasing cardiovascular risk. This integrated mechanism likely explains the stronger association of UAI with CAD and mortality, particularly among women [50].

### 4.1 Strength and Limitations

This study is among the first to evaluate the predictive value of the UAI in combination with SUA in a Middle Eastern population. Its strengths include a large sample size, prospective cohort design, and long‐term follow‐up, allowing for robust assessment of cardiovascular outcomes. Additionally, gender‐stratified analyses provided valuable insights into sex‐specific differences.

However, several limitations should be acknowledged. The study did not differentiate between pre‐menopausal and post‐menopausal women, despite evidence suggesting hormonal influences on SUA levels [51]. Furthermore, the analysis focused primarily on atherosclerosis‐related outcomes, and future studies are needed to explore the role of UA‐related indices in other chronic diseases. Another limitation of this study is the lack of detailed information on medication use and renal function at baseline. Medication data were limited to general categories without details on type or dosage, and renal status was based on self‐report without objective measures such as GFR. Therefore, potential confounding effects of medications and renal function could not be fully adjusted for, which may have introduced residual confounding.

## 5 Conclusion

In this 10‐year prospective cohort study, we compared the predictive value of the UAI and SUA for cardiovascular outcomes. Our findings demonstrated that while SUA showed only a modest association with CAD in the overall population, the UAI was a significantly stronger and more consistent predictor of CAD and mortality outcomes. Notably, the predictive performance of UAI was superior across both sexes, with a more pronounced effect observed in women. In contrast, neither SUA nor UAI showed significant associations with cerebrovascular events, suggesting limited utility of these markers in stroke prediction. The enhanced performance of UAI may be attributed to its ability to integrate key metabolic components, including TGs and FBG, alongside UA, thereby providing a more comprehensive assessment of cardiometabolic risk.

Overall, the UAI appears to be a more robust and clinically useful biomarker than SUA alone for predicting CAD and mortality, particularly in women. These findings support the potential application of this composite index in cardiovascular risk stratification and highlight the importance of considering combined metabolic pathways in disease prediction.

## Author Contributions

S.D. and H.E. conducted the statistical analysis. F.K. and M.I. contributed to the conceptualization and wrote the original draft. B.S., H.A., H.M., A.R., M.S., M.H., and A.H.‐B. were responsible for data curation. M.M. and M.G.‐M. provided supervision and scientific consultation. All authors reviewed and approved the final manuscript.

## Ethics Statement

Accordingly, the study protocol was validated by the Ethics Committee of the Mashhad University of Medical Sciences (MUMS) (ethical approval code: IR.MUMS.IRH.REC.1403.133) and the Institutional Review Board of Mashhad University Medical Center.

## Consent

Informed consent was obtained from all subjects.

## Conflicts of Interest

The authors declare no conflicts of interest.

## Data Availability

The data sets used and/or analyzed during the current study are available from the corresponding author on reasonable request.
